# Bilateral rhabdoid meningioma mimicking glioma: an unusual occurrence

**DOI:** 10.3205/000253

**Published:** 2017-08-14

**Authors:** Varsha Dalal, Fouzia Siraj, Manveen Kaur, K.B. Shankar, Avninder Singh

**Affiliations:** 1 National Institute of Pathology, Indian Council of Medical Research (ICMR), New Delhi, India; 2 Department of Neurosurgery, Safdarjung Hospital, New Delhi, India

**Keywords:** bilateral, meningioma, rhabdoid

## Abstract

Rhabdoid meningioma is an infrequent variant of meningioma, introduced for the first time in the 2000 World Health Organization’s classification of tumors of the nervous system. Owing to its aggressive clinical course and high proliferative index, it has been classified as a grade III neoplasm. We describe a fifty-year-old male with headache, weakness of limbs, and altered sensorium. CT showed hetero-dense enhancing mass lesions in both right and left parietal areas raising suspicion of high grade glioma. Histopathologic and immunohistochemical examination revealed a tumor with features of rhabdoid meningioma. A review of literature did not reveal any bilateral occurrence of this tumor.

## Zusammenfassung

Das rhabdoide Meningeom ist eine seltene Meningeom-Variante, die erstmals im Jahr 2000 in die WHO-Klassifikation von Tumoren des zentralen Nervensystems aufgenommen wurde. Aufgrund ihrer Aggressivität und hohen Proliferation wurde sie als Grad III-Neoplasie klassifiziert. Wir berichten über einen 50-jährigen Mann mit Kopfschmerzen, Schwäche der Extremitäten und verändertem Empfindungsvermögen. Das CT zeigte heterodense, sich vergrößernde Läsionen sowohl in der rechten als auch in der linken Parietalregion, die auf ein hochgradiges Gliom hinwiesen. Histopathologische und immunhistochemische Untersuchungen ließen einen Tumor mit Eigenschaften eines rhabdoiden Meningeoms erkennen. In der ausgewerteten Fachliteratur konnte kein Fall eines beidseitig auftretenden Tumors gefunden werden.

## Introduction

Meningiomas are tumors of meningothelial origin having a wide range of histological appearances. They are most commonly benign and slow growing in nature. Some subtypes however are recognized to display aggressive behavior including atypical, clear cell, chordoid meningiomas (WHO grade II) and papillary and anaplastic variants belonging to WHO grade III [1], [2]. Recently, rhabdoid meningiomas have been added to the grade III subtype. These anaplastic meningiomas represent 1–2% of all meningiomas. They are associated with rapid growth, brain invasion and frequent recurrences [3], [4]. 

## Case description

**Figure 1:**
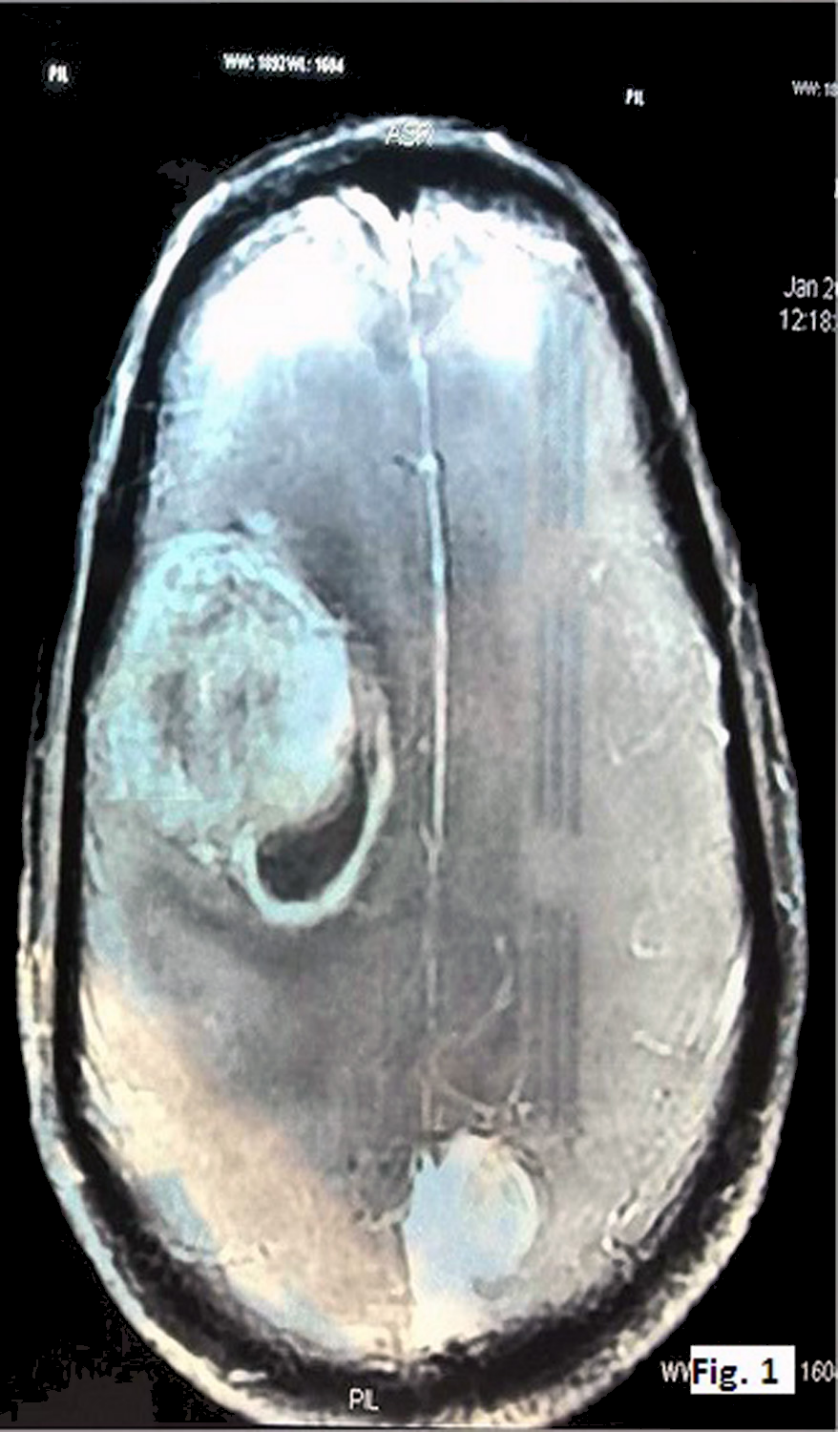
CT image showing hetero-dense lesions in both right and left parietal areas

**Figure 2:**
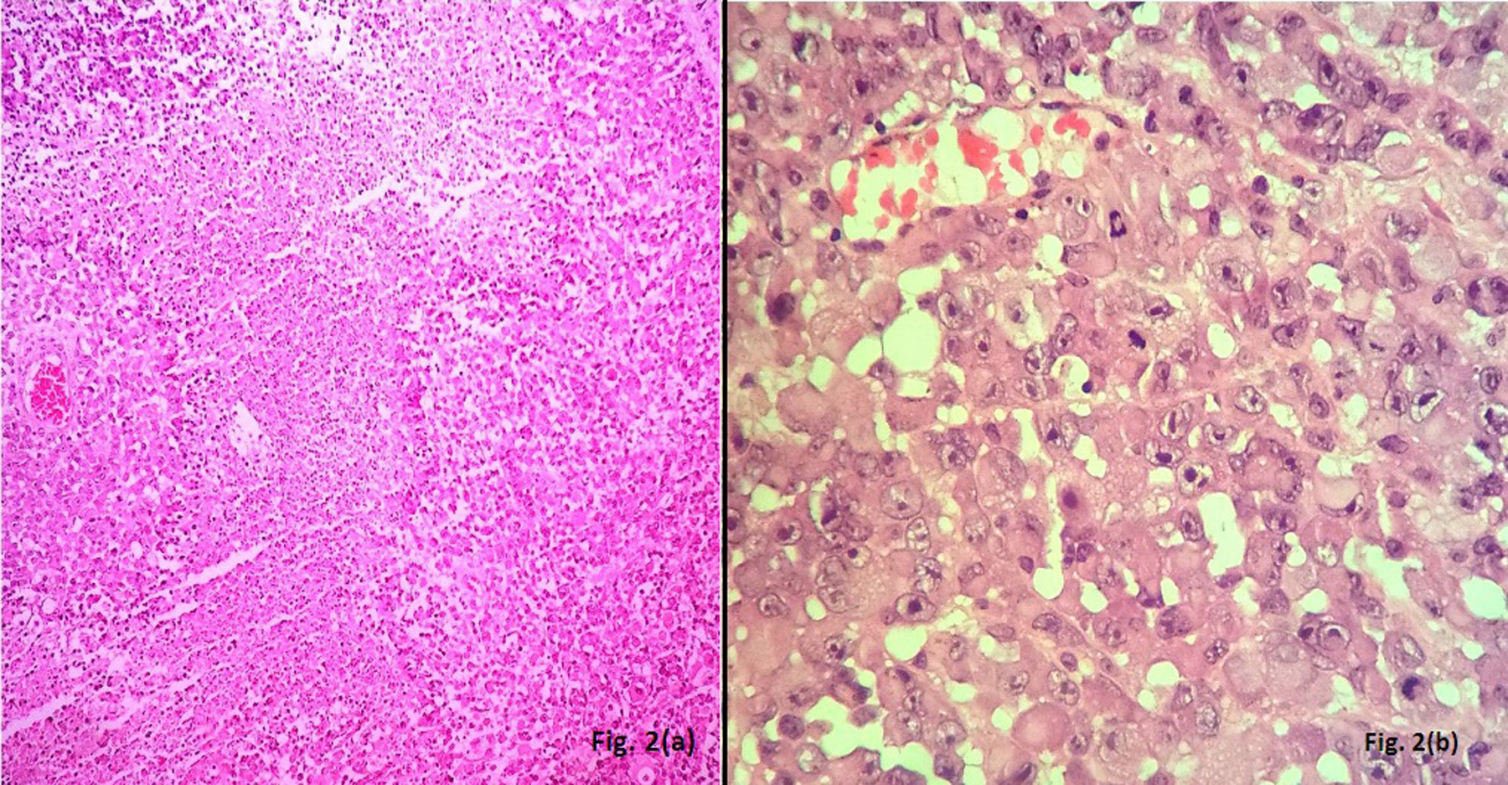
Figure 2(a): Section showing tumor with large areas of necrosis (H&E 40X); (b): Large polygonal cells with abundant eosinophilic cytoplasm, eccentric nuclei, prominent nucleoli and brisk mitoses (H&E 400X)

**Figure 3:**
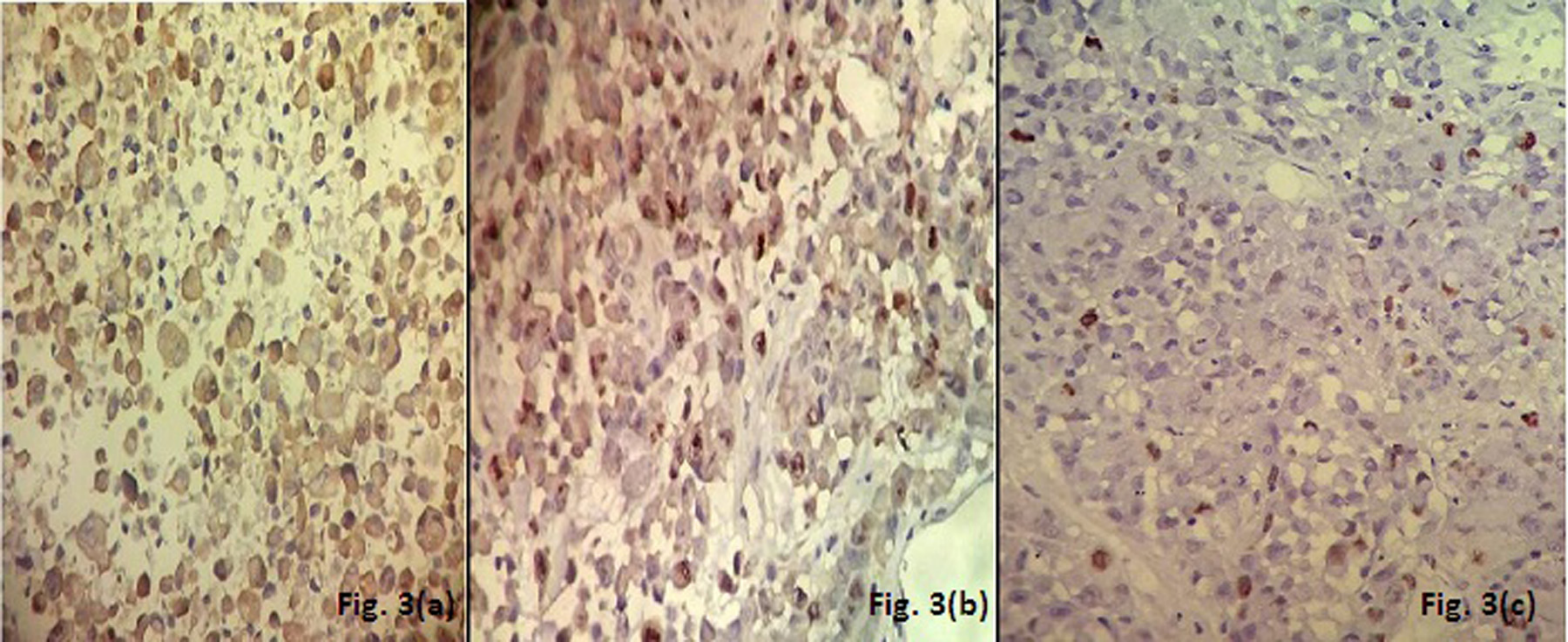
Immunohistochemistry showing positivity for (a): EMA, (b): D2-40, (c): Ki67

A 50-year-old male presented with headache since the past two months. He also complained of weakness in both upper and lower limbs and altered sensorium for the past fifteen days. On physical examination, the patient was conscious with a GCS of E4M6V4. The power was reduced on the left side (3/5), but maintained on the right side (5/5). Computed tomography (CT) revealed well defined hetero-dense masses measuring 5x4 cm and 2x2 cm in the right and the left parietal region respectively with surrounding edema and mass effect (Figure 1 (Fig. 1)). A diagnosis of glioblastoma multiforme was suggested. Total excision of both the masses was done and they were sent for examination. Histopathology of both lesions showed a tumor with cells arranged diffusely in sheets as well as scattered singly. The cells had abundant amounts of eosinophilic cytoplasm, eccentrically placed nuclei, and prominent nucleoli. Areas of high mitotic activity and necrosis were also noted (Figure 2 (Fig. 2)). The possibilities considered were glioblastoma multiforme, metastases, melanoma, plasmacytoma, and rhabdoid meningioma. On IHC (immunohistochemistry), the tumor cells were negative for GFAP, CK, HMB-45 and showed diffuse positivity for D2-40 and vimentin. Focal positivity for EMA was also observed. The high mitotic activity was highlighted by Ki67 positivity demonstrating a mean labeling index of 15% (Figure 3 (Fig. 3)). Absence of pigment and HMB-45 reactivity ruled out a melanocytic lesion. Absence of mott cells and CD 138 reactivity ruled out plasmacytoma. Absence of any other primary lesion on imaging and CK negativity eliminated metastatic carcinoma. GFAP negativity excluded the possibility of glioma. Finally, EMA, D2-40, and vimentin positivity confirmed the meningeal nature of the tumor cells. Thus, a final diagnosis of bilateral rhabdoid meningioma was rendered. Often, the presence of classical areas of meningothelial appearance in the tumor help in reaching the diagnosis [1]. However, these were absent in the present case. Postoperatively, there was improvement in the neurological deficit and subsequently, the patient was put on radiotherapy. He received 30 cycles of radiotherapy with a radiation dose of 60 Gy. Till one year follow-up, the patient is asymptomatic and doing well. 

## Discussion

Meningiomas are neoplasms thought to derive from arachnoidal cap cells in the meningeal coverings of the spinal cord and brain. Meningiomas are generally benign, slow growing tumors which produce neurological symptoms and signs due to their compression of adjacent structures [5]. The incidence of meningiomas in India ranges from 9–15% of all intracranial neoplasms according to a study done by Dr. A. Vincent Thamburaj [6]. Rhabdoid meningiomas were described for the first time in 1998 as an unusual variant with increased proliferative activity. Later, in 2000, these tumors were included in the revised WHO classification of CNS tumors as an aggressive meningioma corresponding to WHO grade III [1], [3]. 

The most commonly affected age groups are the 5^th^ to 7^th^ decades with a marked female preponderance (11:4). The more aggressive subtypes usually present in younger age groups [1]. Our patient, however, was an elderly male. Presenting symptoms include seizures, hemiparesis, and gait disturbance depending on the site of the tumor [1], [5]. 

The term rhabdoid in this tumor refers to the classical morphology of the tumor cell which bears close resemblance to a rhabdomyoblast without true skeletal muscle differentiation [7]. Numerous tumors may exhibit such phenotype viz. glioblastoma multiforme, metastases, melanoma, plasmacytoma. They were therefore considered as differentials in the present case and were subsequently ruled out on the basis of immunohistochemistry. Apart from this typical morphology, rhabdoid meningioma also exhibits a high level of atypia, mitoses and necrosis. Ki67 (MIB-1) is a valuable guide to malignancy in meningioma. The mean labeling index for such tumors is 14.7% ± 9.8% [2], which was 15% in our case. 

Multiplicity in meningiomas attracts a lot of interest because of its relative rarity, unclear aetiology, and the problems related to proper management strategy. Majority of multiple meningiomas (80–90%) are benign and classified as WHO grade I. Anaplastic or malignant meningiomas (WHO grade III) are the rarest (1–3%) [8], [9]. Tomita et al. described multiple meningiomas consisting of fibrous meningioma and anaplastic meningioma [10]. The occurrence of multiple rhabdoid meningioma has not been reported in literature till date. 

Rhabdoid meningiomas are associated with rapid growth, brain invasion, and frequent recurrences [1]. They also show a propensity to metastasize extracranially (7–43%); lung being the most common site, followed by bone, liver, lymph node, and kidneys. Median overall survival (OS) has been reported to be 1.5 years with a 5-year survival ranging from 47% to 61% [11]. Surgery is the sole modality of treatment followed by whole brain radiation therapy (WBRT), usually in doses ranging from 50 to 60 Gy of radiation via conventional fractionation and delivery techniques. Some studies also recommend the use of gamma knife stereotactic radiosurgery in such aggressive cases to prevent recurrence [12]. Good prognostic factors include aggressive gross total resection, de novo status, and location along the convexity/parasagittal intracranial areas [11]. 

## Notes

### Competing interests

The authors declare that they have no competing interests.
